# *tert*-But­yl(2-hy­droxy­eth­yl)aza­nium 4-[(1,3-thia­zol-2-yl­aza­nid­yl)sulfon­yl]aniline

**DOI:** 10.1107/S1600536812034459

**Published:** 2012-08-08

**Authors:** Hadi D. Arman, Trupta Kaulgud, Edward R. T. Tiekink

**Affiliations:** a Department of Chemistry, University of Texas at San Antonio, One UTSA Circle, San Antonio, Texas 78249-0698, USA; b Department of Chemistry, University of Malaya, 50603 Kuala Lumpur, Malaysia

## Abstract

Two pairs of independent cations and anions comprise the asymmetric unit of the title salt, C_6_H_16_NO^+^·C_9_H_8_N_3_O_2_S_2_^−^. The cations are virtually superimposable and each exhibits a *gauche* disposition of the hy­droxy O and ammonium N atoms [the O—C—C—N torsion angles are 55.5 (3) and 57.5 (3)°]. Significant differences are seen in the mol­ecular structures of the anions as seen in the S—N—C—S [1.1 (3) and 32.9 (3)°] and C—S—N—C [−69.7 (2) and 91.4 (2)°] torsion angles. Despite the variations in conformation, intra­molecular hypervalent S⋯O inter­actions persist in each anion [3.078 (2) and 2.8730 (19) Å]. In the crystal, supra­molecular double layers are formed in the *bc* plane, being sustained by O—H⋯N, N—H⋯O and N—H⋯N hydrogen bonding. These are connected along the *a* axis *via* C—H⋯O inter­actions.

## Related literature

For structural studies of sulfathia­zole and derivatives, see: Bingham *et al.* (2001 ▶); Caira (2007 ▶). For previous crystal engineering studies, see: Arman, Kaulgud, Miller, Poplaukhin *et al.* (2012 ▶); Arman, Kaulgud, Miller & Tiekink (2012 ▶). For hypervalent S⋯O inter­actions, see: O’Leary & Wallis (2007 ▶). For the structure of a related aza­nide, see: Brennan *et al.* (1971 ▶).
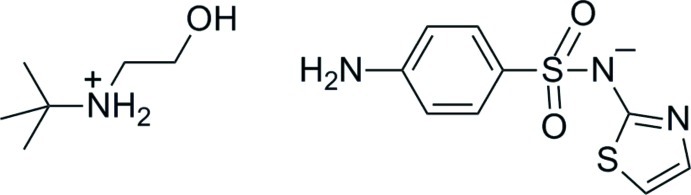


## Experimental

### 

#### Crystal data

C_6_H_16_NO^+^·C_9_H_8_N_3_O_2_S_2_^−^
*M* *_r_* = 372.50
Monoclinic, 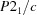

*a* = 13.893 (3) Å
*b* = 11.771 (3) Å
*c* = 22.191 (5) Å
β = 91.401 (4)°
*V* = 3627.9 (15) Å^3^
*Z* = 8
Mo *K*α radiation
μ = 0.32 mm^−1^
*T* = 98 K
0.35 × 0.30 × 0.07 mm

#### Data collection

Rigaku AFC12/SATURN724 diffractometer
18273 measured reflections
8255 independent reflections
7234 reflections with *I* > 2σ(*I*)
*R*_int_ = 0.056
Standard reflections: 0

#### Refinement

*R*[*F* ^2^ > 2σ(*F* ^2^)] = 0.055
*wR*(*F* ^2^) = 0.136
*S* = 1.08
8255 reflections
451 parameters
8 restraints
H atoms treated by a mixture of independent and constrained refinement
Δρ_max_ = 0.36 e Å^−3^
Δρ_min_ = −0.45 e Å^−3^

### 

Data collection: *CrystalClear* (Molecular Structure Corporation & Rigaku, 2005 ▶); cell refinement: *CrystalClear*; data reduction: *Crystal­Clear*; program(s) used to solve structure: *SHELXS97* (Sheldrick, 2008 ▶); program(s) used to refine structure: *SHELXL97* (Sheldrick, 2008 ▶); molecular graphics: *ORTEP-3 for Windows* (Farrugia, 1997 ▶), *DIAMOND* (Brandenburg, 2006 ▶) and *QMol* (Gans & Shalloway, 2001 ▶); software used to prepare material for publication: *publCIF* (Westrip, 2010 ▶).

## Supplementary Material

Crystal structure: contains datablock(s) global, I. DOI: 10.1107/S1600536812034459/bt5992sup1.cif

Structure factors: contains datablock(s) I. DOI: 10.1107/S1600536812034459/bt5992Isup2.hkl

Supplementary material file. DOI: 10.1107/S1600536812034459/bt5992Isup3.cml

Additional supplementary materials:  crystallographic information; 3D view; checkCIF report

## Figures and Tables

**Table 1:** Hydrogen-bond geometry (Å, °)

*D*—H⋯*A*	*D*—H	H⋯*A*	*D*⋯*A*	*D*—H⋯*A*
O21—H21*o*⋯N1	0.84 (3)	1.90 (3)	2.739 (3)	177 (2)
O31—H31*o*⋯N2	0.83 (1)	1.97 (1)	2.796 (3)	174 (3)
N3—H2*n*⋯O12^i^	0.88 (2)	2.19 (2)	3.027 (3)	161 (3)
N13—H11*n*⋯O2^ii^	0.89 (2)	2.34 (2)	3.201 (3)	164 (2)
N13—H12*n*⋯O1^iii^	0.88 (2)	2.25 (2)	3.098 (3)	160 (2)
N21—H21*n*⋯N12	0.92	1.91	2.832 (3)	178
N21—H22*n*⋯O31	0.92	1.94	2.862 (3)	176
N31—H31*n*⋯N11	0.92	1.89	2.802 (3)	175
N31—H32*n*⋯O21	0.92	2.02	2.934 (3)	174
C2—H2⋯O11^iv^	0.95	2.43	3.356 (3)	164
C6—H6⋯O12^i^	0.95	2.50	3.263 (3)	137

Symmetry codes: (i) 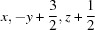; (ii) 
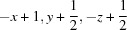
; (iii) 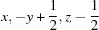; (iv) 
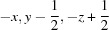
.

**Fig. 1.**
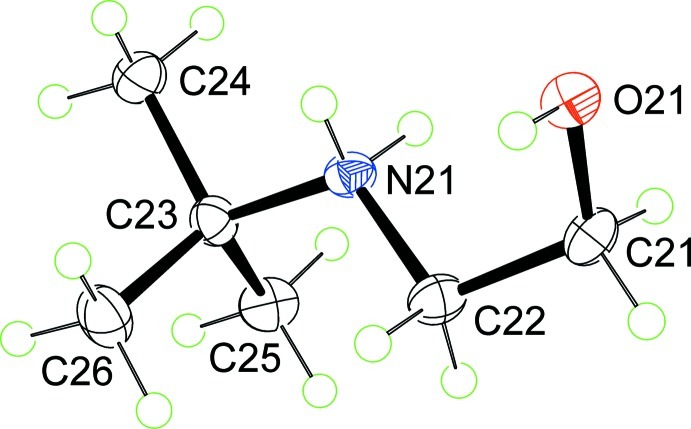
Molecular structure of the first independent cation of (I) showing atom-labelling scheme and displacement ellipsoids at the 50% probability level.

**Fig. 2.**
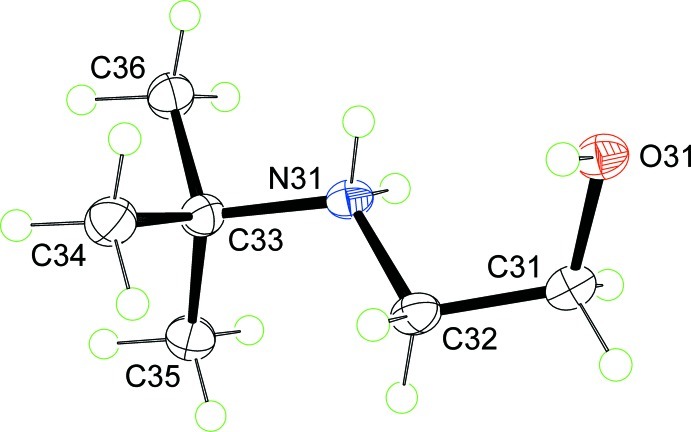
Molecular structure of the second independent cation of (I) showing atom-labelling scheme and displacement ellipsoids at the 50% probability level.

**Fig. 3.**
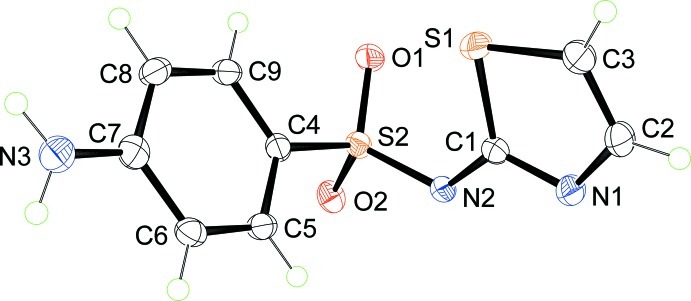
Molecular structure of the first independent anion of (I) showing atom-labelling scheme and displacement ellipsoids at the 50% probability level.

**Fig. 4.**
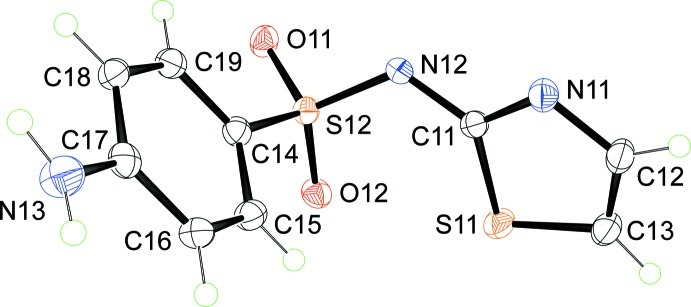
Molecular structure of the second independent anion of (I) showing atom-labelling scheme and displacement ellipsoids at the 50% probability level.

**Fig. 5.**
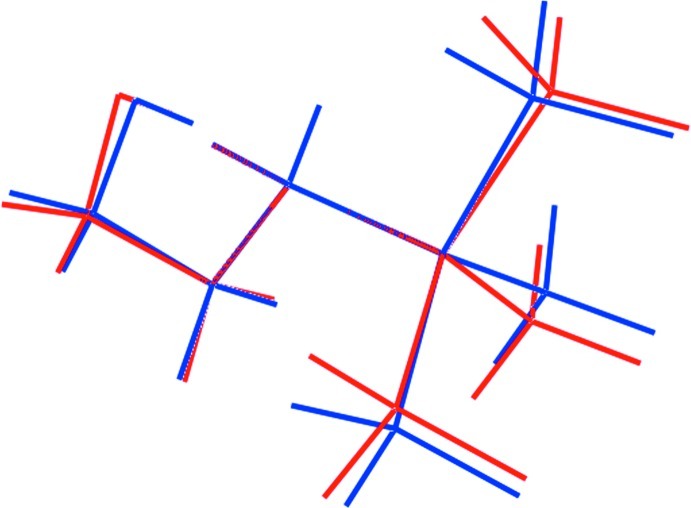
Superimposition of the two independent cations in (I). The C—N—C fragments have been superimposed. The N21 and and N31-containing molecules are shown as red and blue images, respectively.

**Fig. 6.**
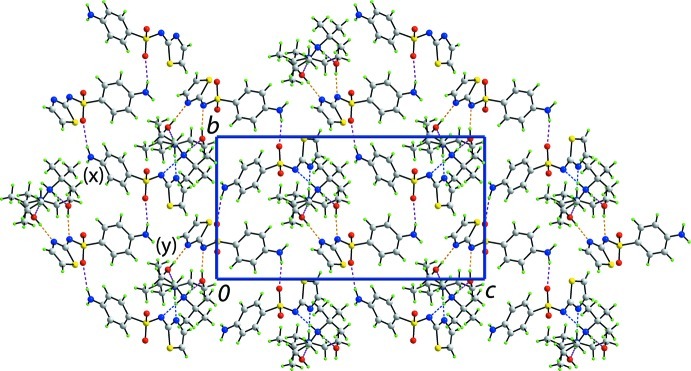
Supramolecular layer in the bc plane in (I). The arrays are mediated by O–H···N, N—H···O and N—H···N hydrogen bonds which are shown as orange, purple and blue dashed lines, respectively. See text for the meaning of (x) and (y).

**Fig. 7.**
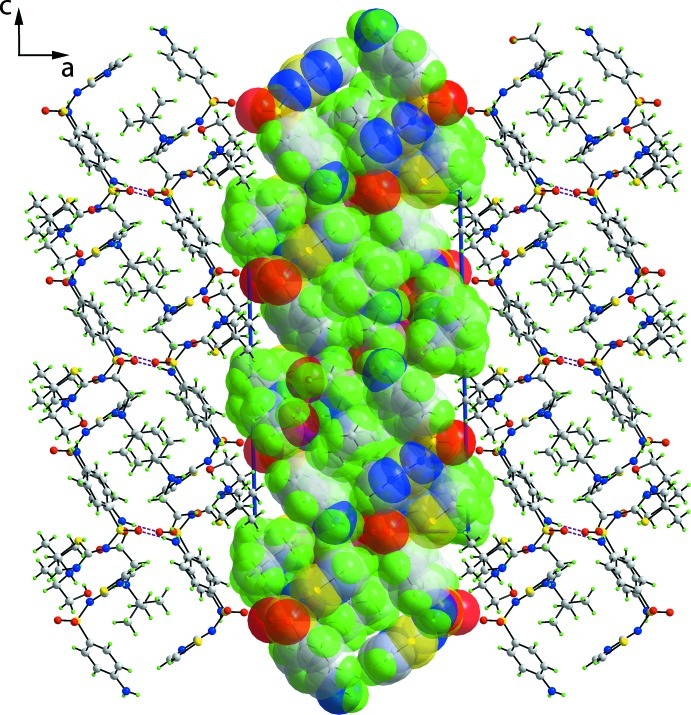
Unit-cell contents in (I) viewed in projection down the b axis. One double-layer is presented in space-filling mode.

## supplementary crystallographic information

### Comment

Sulfathiazole and derivatives are well known to form polymorphs, co-crystals and solvates (Bingham *et al.*, 2001; Caira, 2007) and attracted our interest in the context of previous crystal engineering studies conducted by our group (Arman, Kaulgud, Miller, Poplaukhin *et al.*, 2012; Arman, Kaulgud, Miller & Tiekink, 2012). Herein, the crystal and molecular structure of the title salt, (I), is described.

Two independent cations, Figs 1 and 2, and two independent anions, Figs 3 and 4, comprise the asymmetric unit of (I). The cations are virtually super-imposable, Fig. 5. The key feature of the molecular structure of each cation is the *gauche*-disposition of the hydroxyl-O and ammonium-N atoms as seen in the values of the O21—C21—C22—N21 and O31—C31—C32—N31 torsion angles of 55.5 (3) and 57.5 (3)°, respectively A greater disparity is noted in the molecular structures of the anions. Thus, while the S2—N2—C1—S1 torsion angle is 1.1 (3)°, the comparable S12—N12—C11—S11 angle of 32.9 (3)° indicates a significant twist. There is also a twist about the S—N bond as seen in the values of the C4—S2—N2—C1 and C14—S12—N12—C11 torsion angles of -69.7 (2) and 91.4 (2)°, respectively. The dihedral angle between the respective five- and six-membered rings in each molecule is 86.46 (12) and 76.91 (11)°. Despite the variations in orientation, intramolecular hypervalent S···O interactions (O'Leary & Wallis, 2007) persist, *i.e*. 3.078 (2) and 2.8730 (19) Å, respectively. A similar contact of 3.01 Å was noted previously for the azanide anion isolated as its (4-sulfamoylphenyl)methanaminium salt (Brennan *et al.*, 1971).

As expected from the composition, significant hydrogen bonding is apparent in the crystal structure of (I). Thus, O—H···N, N—H···O and N—H···N interactions lead to supramolecular double layers in the *bc* plane, Fig. 6 and Table 1. Both of the independent hydroxyl groups hydrogen bonds to a single anion, *i.e*. O21—H to the thiazole-N1, and O31—H to the azanide-N2. The ammonium cations are connected to each other *via N*—*H*···*O*(hydroxyl) hydrogen bonds with the remaining ammonium-H atom on each cation connected to the same anion, *via* N21—*H*···N12-azanide and N31–*H*···N11(thiazole) hydrogen bonds. This results in the formation of two cation plus two anion aggregates. These are connected into a two dimensional sheet *via* aniline-*N*–*H*···*O*(sulfonyl) hydrogen bonds. The sheet thus formed is connected into a double layer *via* aniline-N31–*H*···O2(sulfonyl) hydrogen bonds, indicated by '(*x*)' in Fig. 6, leading to 12-membered {···HNH···O═S═O···}_2_ synthons. The N3—H1n atom, labelled with '(*y*)' in Fig. 6, does not participate in a formal hydrogen bond. The closest potential acceptor atom, *i.e*. a symmetry-related O21(hydroxyl), is proximate at 3.472 (3) Å but, the intervention of a methyl group precludes the formation of a significant hydrogen bonding interaction. In addition to C–H···O interactions that contribute to the stability of the double layer, further C2–H···O11 contacts occur between layers that stack along the *a* direction, Fig. 7.

### Experimental

Sulfathiazole (Sigma-Aldrich) and *tert*-butyl(2-hydroxyethyl)amine (Sigma-Aldrich) were used as delivered. Single crystals of (I) were harvested from a 1:1 methanol/tetrahydrofuran (10 ml) solution of the amine and a stoichiometric amount of sulfathiazole (25 mg) that had been let to stand for about a week; *M*.pt 443–448 K.

### Refinement

C-bound H-atoms were placed in calculated positions (C—H 0.95–0.99 Å) and were included in the refinement in the riding model approximation with *U*_iso_(H) set to 1.2–1.5*U*_eq_(C). The ammonium-H atoms were included in their idealized positions with N—H = 0.92 Å and with *U*_iso_(H) set to 1.5*U*_eq_(N). The O– and remaining N-bound H-atoms were located in a difference Fourier map and refined with O—H = 0.84±0.01 Å and N—H = 0.88±0.01 Å (with H···H = 1.52±0.02 Å), and with *U*_iso_(H) = 1.2(N)-1.5(O)*U*_eq_(N, O).

### Crystal data

**Table:**

C_6_H_16_NO^+^·C_9_H_8_N_3_O_2_S_2_^−^	*F*(000) = 1584
*M**_r_* = 372.50	*D*_x_ = 1.364 Mg m^−^^3^
Monoclinic, *P*2_1_/*c*	Mo *K*α radiation, λ = 0.71073 Å
Hall symbol: -P 2ybc	Cell parameters from 24162 reflections
*a* = 13.893 (3) Å	θ = 2.4–40.3°
*b* = 11.771 (3) Å	µ = 0.32 mm^−^^1^
*c* = 22.191 (5) Å	*T* = 98 K
β = 91.401 (4)°	Plate, colourless
*V* = 3627.9 (15) Å^3^	0.35 × 0.30 × 0.07 mm
*Z* = 8	

### Data collection

**Table:**

Rigaku AFC12K/SATURN724 diffractometer	7234 reflections with *I* > 2σ(*I*)
Radiation source: fine-focus sealed tube	*R*_int_ = 0.056
Graphite monochromator	θ_max_ = 27.5°, θ_min_ = 2.4°
ω scans	*h* = −17→18
18273 measured reflections	*k* = −15→5
8255 independent reflections	*l* = −23→28

### Refinement

**Table:**

Refinement on *F*^2^	Primary atom site location: structure-invariant direct methods
Least-squares matrix: full	Secondary atom site location: difference Fourier map
*R*[*F*^2^ > 2σ(*F*^2^)] = 0.055	Hydrogen site location: inferred from neighbouring sites
*wR*(*F*^2^) = 0.136	H atoms treated by a mixture of independent and constrained refinement
*S* = 1.08	*w* = 1/[σ^2^(*F*_o_^2^) + (0.0435*P*)^2^ + 3.8739*P*] where *P* = (*F*_o_^2^ + 2*F*_c_^2^)/3
8255 reflections	(Δ/σ)_max_ = 0.001
451 parameters	Δρ_max_ = 0.36 e Å^−^^3^
8 restraints	Δρ_min_ = −0.45 e Å^−^^3^

### Special details

**Table:**

Geometry. All s.u.'s (except the s.u. in the dihedral angle between two l.s. planes) are estimated using the full covariance matrix. The cell s.u.'s are taken into account individually in the estimation of s.u.'s in distances, angles and torsion angles; correlations between s.u.'s in cell parameters are only used when they are defined by crystal symmetry. An approximate (isotropic) treatment of cell s.u.'s is used for estimating s.u.'s involving l.s. planes.
Refinement. Refinement of *F*^2^ against ALL reflections. The weighted *R*-factor *wR* and goodness of fit *S* are based on *F*^2^, conventional *R*-factors *R* are based on *F*, with *F* set to zero for negative *F*^2^. The threshold expression of *F*^2^ > 2σ(*F*^2^) is used only for calculating *R*-factors(gt) *etc*. and is not relevant to the choice of reflections for refinement. *R*-factors based on *F*^2^ are statistically about twice as large as those based on *F*, and *R*- factors based on ALL data will be even larger.

### Fractional atomic coordinates and isotropic or equivalent isotropic displacement parameters (Å^2^)

**Table:**

	*x*	*y*	*z*	*U*_iso_*/*U*_eq_	
S1	0.16821 (5)	0.10093 (5)	0.47147 (3)	0.02469 (14)	
S2	0.36615 (4)	0.23512 (5)	0.50339 (3)	0.01958 (13)	
O1	0.38569 (13)	0.11406 (15)	0.50058 (8)	0.0254 (4)	
O2	0.45054 (12)	0.30808 (16)	0.50206 (8)	0.0243 (4)	
N1	0.15549 (15)	0.26042 (19)	0.39238 (10)	0.0236 (4)	
N2	0.29650 (14)	0.27894 (17)	0.45040 (9)	0.0192 (4)	
N3	0.16937 (17)	0.3130 (2)	0.73461 (10)	0.0282 (5)	
H1n	0.174 (2)	0.261 (2)	0.7627 (11)	0.042*	
H2n	0.153 (2)	0.3833 (12)	0.7421 (14)	0.042*	
C1	0.21311 (17)	0.2228 (2)	0.43601 (11)	0.0196 (5)	
C2	0.07372 (18)	0.1937 (2)	0.38592 (12)	0.0261 (5)	
H2	0.0251	0.2092	0.3562	0.031*	
C3	0.06738 (19)	0.1054 (2)	0.42428 (12)	0.0269 (5)	
H3	0.0152	0.0533	0.4252	0.032*	
C4	0.30798 (17)	0.2583 (2)	0.57212 (10)	0.0187 (4)	
C5	0.26187 (17)	0.3620 (2)	0.58306 (11)	0.0203 (5)	
H5	0.2614	0.4196	0.5531	0.024*	
C6	0.21712 (17)	0.3812 (2)	0.63700 (11)	0.0211 (5)	
H6	0.1866	0.4520	0.6441	0.025*	
C7	0.21672 (17)	0.2954 (2)	0.68182 (11)	0.0221 (5)	
C8	0.26184 (18)	0.1920 (2)	0.67037 (11)	0.0236 (5)	
H8	0.2615	0.1336	0.6999	0.028*	
C9	0.30725 (18)	0.1737 (2)	0.61606 (11)	0.0221 (5)	
H9	0.3380	0.1030	0.6088	0.027*	
S11	0.28142 (5)	0.98598 (5)	0.32531 (3)	0.02459 (14)	
S12	0.14994 (4)	0.80923 (5)	0.23655 (3)	0.01828 (13)	
O11	0.06213 (12)	0.74301 (16)	0.23845 (8)	0.0230 (4)	
O12	0.13976 (12)	0.93223 (15)	0.23321 (8)	0.0224 (4)	
N11	0.35743 (15)	0.79060 (18)	0.34885 (9)	0.0222 (4)	
N12	0.21478 (14)	0.76971 (17)	0.29279 (9)	0.0197 (4)	
N13	0.37494 (17)	0.6429 (2)	0.02991 (11)	0.0298 (5)	
H11n	0.4289 (15)	0.676 (2)	0.0189 (15)	0.045*	
H12n	0.364 (2)	0.5731 (14)	0.0170 (15)	0.045*	
C11	0.28445 (16)	0.8372 (2)	0.31912 (10)	0.0174 (4)	
C12	0.41351 (18)	0.8728 (2)	0.37772 (11)	0.0235 (5)	
H12	0.4690	0.8536	0.4015	0.028*	
C13	0.38494 (19)	0.9815 (2)	0.37019 (11)	0.0258 (5)	
H13	0.4169	1.0459	0.3870	0.031*	
C14	0.21076 (17)	0.7648 (2)	0.17170 (10)	0.0189 (4)	
C15	0.28885 (17)	0.8281 (2)	0.15081 (11)	0.0214 (5)	
H15	0.3047	0.8988	0.1691	0.026*	
C16	0.34297 (18)	0.7878 (2)	0.10361 (11)	0.0223 (5)	
H16	0.3954	0.8316	0.0897	0.027*	
C17	0.32109 (17)	0.6825 (2)	0.07608 (11)	0.0212 (5)	
C18	0.24137 (17)	0.6213 (2)	0.09704 (11)	0.0213 (5)	
H18	0.2243	0.5511	0.0785	0.026*	
C19	0.18755 (17)	0.6616 (2)	0.14420 (11)	0.0206 (5)	
H19	0.1345	0.6186	0.1579	0.025*	
O21	0.20607 (12)	0.43422 (15)	0.31746 (8)	0.0221 (4)	
H21o	0.190 (2)	0.383 (2)	0.3413 (12)	0.033*	
N21	0.12174 (14)	0.62867 (17)	0.37680 (9)	0.0190 (4)	
H21n	0.1508	0.6739	0.3487	0.023*	
H22n	0.1699	0.5903	0.3974	0.023*	
C21	0.11863 (18)	0.4853 (2)	0.29455 (11)	0.0220 (5)	
H21A	0.0783	0.4260	0.2748	0.026*	
H21B	0.1347	0.5423	0.2636	0.026*	
C22	0.06114 (17)	0.5426 (2)	0.34340 (11)	0.0228 (5)	
H22A	0.0041	0.5805	0.3249	0.027*	
H22B	0.0383	0.4846	0.3720	0.027*	
C23	0.07187 (17)	0.7065 (2)	0.42153 (11)	0.0203 (5)	
C24	0.15194 (18)	0.7693 (2)	0.45610 (12)	0.0257 (5)	
H24A	0.1885	0.8158	0.4280	0.039*	
H24B	0.1951	0.7141	0.4758	0.039*	
H24C	0.1236	0.8185	0.4866	0.039*	
C25	0.00759 (19)	0.7910 (2)	0.38651 (12)	0.0268 (5)	
H25A	0.0471	0.8361	0.3595	0.040*	
H25B	−0.0243	0.8415	0.4149	0.040*	
H25C	−0.0412	0.7492	0.3628	0.040*	
C26	0.0141 (2)	0.6337 (2)	0.46453 (12)	0.0293 (6)	
H26A	−0.0371	0.5939	0.4418	0.044*	
H26B	−0.0146	0.6824	0.4951	0.044*	
H26C	0.0568	0.5780	0.4843	0.044*	
O31	0.26830 (12)	0.51401 (15)	0.44630 (8)	0.0220 (4)	
H31o	0.275 (2)	0.4438 (9)	0.4450 (14)	0.033*	
N31	0.38695 (14)	0.55543 (17)	0.34314 (9)	0.0184 (4)	
H31n	0.3735	0.6319	0.3438	0.022*	
H32n	0.3293	0.5178	0.3378	0.022*	
C31	0.36309 (17)	0.5610 (2)	0.45343 (11)	0.0224 (5)	
H31A	0.3912	0.5372	0.4929	0.027*	
H31B	0.3588	0.6450	0.4533	0.027*	
C32	0.42873 (17)	0.5231 (2)	0.40364 (11)	0.0220 (5)	
H32A	0.4928	0.5589	0.4094	0.026*	
H32B	0.4373	0.4396	0.4056	0.026*	
C33	0.44814 (17)	0.5317 (2)	0.28840 (11)	0.0209 (5)	
C34	0.46354 (19)	0.4039 (2)	0.28245 (12)	0.0271 (5)	
H34A	0.5006	0.3761	0.3176	0.041*	
H34B	0.4989	0.3881	0.2457	0.041*	
H34C	0.4010	0.3655	0.2803	0.041*	
C35	0.54298 (18)	0.5966 (2)	0.29574 (12)	0.0282 (6)	
H35A	0.5796	0.5666	0.3306	0.042*	
H35B	0.5295	0.6774	0.3021	0.042*	
H35C	0.5807	0.5873	0.2593	0.042*	
C36	0.39072 (18)	0.5777 (2)	0.23432 (11)	0.0241 (5)	
H36A	0.3817	0.6598	0.2390	0.036*	
H36B	0.3277	0.5402	0.2319	0.036*	
H36C	0.4258	0.5626	0.1974	0.036*	

### Atomic displacement parameters (Å^2^)

**Table:**

	*U*^11^	*U*^22^	*U*^33^	*U*^12^	*U*^13^	*U*^23^
S1	0.0275 (3)	0.0196 (3)	0.0267 (3)	−0.0048 (2)	−0.0058 (2)	0.0056 (2)
S2	0.0187 (3)	0.0186 (3)	0.0213 (3)	0.0015 (2)	−0.0034 (2)	−0.0021 (2)
O1	0.0311 (10)	0.0188 (9)	0.0260 (9)	0.0069 (7)	−0.0054 (7)	−0.0034 (7)
O2	0.0177 (8)	0.0286 (10)	0.0264 (9)	−0.0019 (7)	−0.0026 (7)	−0.0046 (7)
N1	0.0230 (10)	0.0228 (11)	0.0246 (11)	−0.0042 (8)	−0.0055 (8)	0.0027 (8)
N2	0.0202 (10)	0.0170 (10)	0.0203 (10)	−0.0020 (7)	−0.0037 (7)	−0.0004 (8)
N3	0.0342 (12)	0.0267 (12)	0.0240 (11)	−0.0049 (9)	0.0045 (9)	0.0014 (9)
C1	0.0225 (11)	0.0168 (11)	0.0195 (11)	0.0015 (9)	−0.0010 (9)	−0.0024 (9)
C2	0.0233 (12)	0.0278 (13)	0.0267 (13)	−0.0049 (10)	−0.0076 (10)	0.0011 (10)
C3	0.0265 (13)	0.0254 (13)	0.0285 (13)	−0.0077 (10)	−0.0048 (10)	0.0020 (10)
C4	0.0204 (11)	0.0187 (11)	0.0169 (11)	0.0009 (8)	−0.0015 (8)	−0.0024 (9)
C5	0.0212 (11)	0.0168 (11)	0.0229 (11)	0.0000 (9)	−0.0021 (9)	0.0017 (9)
C6	0.0208 (11)	0.0176 (11)	0.0247 (12)	−0.0008 (8)	−0.0030 (9)	−0.0018 (9)
C7	0.0177 (11)	0.0250 (13)	0.0232 (12)	−0.0065 (9)	−0.0032 (9)	−0.0002 (10)
C8	0.0254 (12)	0.0201 (12)	0.0249 (12)	−0.0040 (9)	−0.0061 (9)	0.0060 (10)
C9	0.0255 (12)	0.0159 (11)	0.0247 (12)	−0.0005 (9)	−0.0047 (9)	0.0009 (9)
S11	0.0278 (3)	0.0170 (3)	0.0287 (3)	−0.0006 (2)	−0.0042 (2)	−0.0020 (2)
S12	0.0177 (3)	0.0187 (3)	0.0183 (3)	−0.0004 (2)	−0.0012 (2)	0.0003 (2)
O11	0.0186 (8)	0.0276 (10)	0.0228 (9)	−0.0041 (7)	−0.0007 (6)	−0.0013 (7)
O12	0.0260 (9)	0.0192 (9)	0.0220 (8)	0.0040 (7)	−0.0008 (7)	0.0012 (7)
N11	0.0226 (10)	0.0219 (11)	0.0218 (10)	0.0010 (8)	−0.0029 (8)	−0.0012 (8)
N12	0.0224 (10)	0.0185 (10)	0.0181 (10)	−0.0026 (8)	−0.0026 (7)	0.0039 (8)
N13	0.0309 (12)	0.0275 (12)	0.0313 (12)	−0.0018 (9)	0.0072 (9)	−0.0037 (10)
C11	0.0197 (11)	0.0170 (11)	0.0155 (10)	−0.0012 (8)	0.0030 (8)	−0.0012 (8)
C12	0.0229 (12)	0.0264 (13)	0.0209 (12)	−0.0037 (9)	−0.0033 (9)	−0.0032 (10)
C13	0.0277 (13)	0.0265 (13)	0.0230 (12)	−0.0072 (10)	−0.0016 (10)	−0.0036 (10)
C14	0.0195 (11)	0.0185 (11)	0.0186 (11)	0.0014 (8)	−0.0014 (8)	0.0008 (9)
C15	0.0219 (11)	0.0178 (11)	0.0243 (12)	−0.0006 (9)	−0.0015 (9)	0.0002 (9)
C16	0.0233 (12)	0.0203 (12)	0.0234 (12)	−0.0034 (9)	0.0005 (9)	0.0023 (9)
C17	0.0186 (11)	0.0237 (12)	0.0211 (11)	0.0022 (9)	−0.0048 (9)	0.0032 (9)
C18	0.0241 (12)	0.0174 (11)	0.0222 (12)	−0.0009 (9)	−0.0044 (9)	0.0002 (9)
C19	0.0175 (11)	0.0223 (12)	0.0218 (11)	−0.0023 (8)	−0.0024 (9)	0.0038 (9)
O21	0.0238 (9)	0.0189 (9)	0.0236 (9)	−0.0015 (7)	−0.0022 (7)	0.0033 (7)
N21	0.0178 (9)	0.0182 (10)	0.0210 (10)	−0.0027 (7)	−0.0010 (7)	0.0016 (8)
C21	0.0269 (12)	0.0177 (11)	0.0214 (12)	−0.0006 (9)	−0.0047 (9)	−0.0017 (9)
C22	0.0219 (12)	0.0192 (12)	0.0270 (12)	−0.0031 (9)	−0.0023 (9)	−0.0028 (10)
C23	0.0209 (11)	0.0193 (12)	0.0208 (11)	0.0002 (9)	0.0025 (9)	−0.0019 (9)
C24	0.0244 (12)	0.0269 (13)	0.0258 (13)	−0.0027 (10)	−0.0007 (10)	−0.0057 (10)
C25	0.0259 (13)	0.0216 (13)	0.0328 (14)	0.0023 (10)	−0.0010 (10)	−0.0025 (10)
C26	0.0326 (14)	0.0291 (14)	0.0265 (13)	−0.0055 (11)	0.0082 (10)	0.0018 (11)
O31	0.0227 (8)	0.0190 (9)	0.0242 (9)	−0.0016 (7)	−0.0032 (7)	−0.0003 (7)
N31	0.0188 (9)	0.0166 (10)	0.0197 (10)	−0.0011 (7)	−0.0034 (7)	0.0017 (7)
C31	0.0240 (12)	0.0225 (12)	0.0206 (11)	−0.0016 (9)	−0.0036 (9)	−0.0005 (9)
C32	0.0225 (12)	0.0228 (12)	0.0204 (12)	0.0020 (9)	−0.0050 (9)	0.0014 (9)
C33	0.0215 (11)	0.0219 (12)	0.0193 (11)	0.0009 (9)	−0.0013 (9)	−0.0003 (9)
C34	0.0296 (13)	0.0243 (13)	0.0270 (13)	0.0062 (10)	−0.0056 (10)	−0.0019 (10)
C35	0.0208 (12)	0.0343 (15)	0.0294 (13)	−0.0041 (10)	0.0007 (10)	−0.0043 (11)
C36	0.0256 (12)	0.0260 (13)	0.0206 (12)	0.0030 (10)	−0.0009 (9)	0.0019 (10)

### Geometric parameters (Å, º)

**Table:**

S1—C3	1.729 (3)	C18—H18	0.9500
S1—C1	1.758 (3)	C19—H19	0.9500
S2—O1	1.4524 (18)	O21—C21	1.437 (3)
S2—O2	1.4542 (18)	O21—H21O	0.835 (10)
S2—N2	1.590 (2)	N21—C22	1.502 (3)
S2—C4	1.765 (2)	N21—C23	1.529 (3)
N1—C1	1.318 (3)	N21—H21N	0.9200
N1—C2	1.386 (3)	N21—H22N	0.9200
N2—C1	1.365 (3)	C21—C22	1.520 (3)
N3—C7	1.373 (3)	C21—H21A	0.9900
N3—H1N	0.873 (10)	C21—H21B	0.9900
N3—H2N	0.875 (10)	C22—H22A	0.9900
C2—C3	1.348 (4)	C22—H22B	0.9900
C2—H2	0.9500	C23—C26	1.526 (3)
C3—H3	0.9500	C23—C24	1.527 (3)
C4—C9	1.394 (3)	C23—C25	1.535 (3)
C4—C5	1.402 (3)	C24—H24A	0.9800
C5—C6	1.381 (3)	C24—H24B	0.9800
C5—H5	0.9500	C24—H24C	0.9800
C6—C7	1.417 (3)	C25—H25A	0.9800
C6—H6	0.9500	C25—H25B	0.9800
C7—C8	1.395 (4)	C25—H25C	0.9800
C8—C9	1.391 (4)	C26—H26A	0.9800
C8—H8	0.9500	C26—H26B	0.9800
C9—H9	0.9500	C26—H26C	0.9800
S11—C13	1.730 (3)	O31—C31	1.434 (3)
S11—C11	1.757 (2)	O31—H31O	0.833 (10)
S12—O11	1.4493 (17)	N31—C32	1.499 (3)
S12—O12	1.4563 (18)	N31—C33	1.525 (3)
S12—N12	1.591 (2)	N31—H31N	0.9200
S12—C14	1.766 (2)	N31—H32N	0.9200
N11—C11	1.316 (3)	C31—C32	1.517 (3)
N11—C12	1.389 (3)	C31—H31A	0.9900
N12—C11	1.372 (3)	C31—H31B	0.9900
N13—C17	1.365 (3)	C32—H32A	0.9900
N13—H11N	0.882 (10)	C32—H32B	0.9900
N13—H12N	0.882 (10)	C33—C36	1.524 (3)
C12—C13	1.348 (4)	C33—C34	1.525 (3)
C12—H12	0.9500	C33—C35	1.528 (3)
C13—H13	0.9500	C34—H34A	0.9800
C14—C19	1.394 (3)	C34—H34B	0.9800
C14—C15	1.404 (3)	C34—H34C	0.9800
C15—C16	1.388 (3)	C35—H35A	0.9800
C15—H15	0.9500	C35—H35B	0.9800
C16—C17	1.412 (4)	C35—H35C	0.9800
C16—H16	0.9500	C36—H36A	0.9800
C17—C18	1.410 (3)	C36—H36B	0.9800
C18—C19	1.385 (3)	C36—H36C	0.9800
			
C3—S1—C1	89.73 (12)	C22—N21—H22N	107.8
O1—S2—O2	115.27 (11)	C23—N21—H22N	107.8
O1—S2—N2	113.42 (11)	H21N—N21—H22N	107.2
O2—S2—N2	105.65 (11)	O21—C21—C22	112.8 (2)
O1—S2—C4	106.20 (11)	O21—C21—H21A	109.0
O2—S2—C4	108.22 (11)	C22—C21—H21A	109.0
N2—S2—C4	107.80 (11)	O21—C21—H21B	109.0
C1—N1—C2	111.5 (2)	C22—C21—H21B	109.0
C1—N2—S2	120.90 (17)	H21A—C21—H21B	107.8
C7—N3—H1N	118 (2)	N21—C22—C21	110.69 (19)
C7—N3—H2N	116 (2)	N21—C22—H22A	109.5
H1N—N3—H2N	123 (2)	C21—C22—H22A	109.5
N1—C1—N2	120.4 (2)	N21—C22—H22B	109.5
N1—C1—S1	112.79 (18)	C21—C22—H22B	109.5
N2—C1—S1	126.79 (18)	H22A—C22—H22B	108.1
C3—C2—N1	116.0 (2)	C26—C23—C24	110.2 (2)
C3—C2—H2	122.0	C26—C23—N21	108.8 (2)
N1—C2—H2	122.0	C24—C23—N21	106.28 (19)
C2—C3—S1	109.96 (19)	C26—C23—C25	111.8 (2)
C2—C3—H3	125.0	C24—C23—C25	110.4 (2)
S1—C3—H3	125.0	N21—C23—C25	109.06 (19)
C9—C4—C5	119.3 (2)	C23—C24—H24A	109.5
C9—C4—S2	120.35 (18)	C23—C24—H24B	109.5
C5—C4—S2	120.34 (18)	H24A—C24—H24B	109.5
C6—C5—C4	120.7 (2)	C23—C24—H24C	109.5
C6—C5—H5	119.7	H24A—C24—H24C	109.5
C4—C5—H5	119.7	H24B—C24—H24C	109.5
C5—C6—C7	120.1 (2)	C23—C25—H25A	109.5
C5—C6—H6	120.0	C23—C25—H25B	109.5
C7—C6—H6	120.0	H25A—C25—H25B	109.5
N3—C7—C8	120.9 (2)	C23—C25—H25C	109.5
N3—C7—C6	120.1 (2)	H25A—C25—H25C	109.5
C8—C7—C6	119.0 (2)	H25B—C25—H25C	109.5
C9—C8—C7	120.6 (2)	C23—C26—H26A	109.5
C9—C8—H8	119.7	C23—C26—H26B	109.5
C7—C8—H8	119.7	H26A—C26—H26B	109.5
C8—C9—C4	120.4 (2)	C23—C26—H26C	109.5
C8—C9—H9	119.8	H26A—C26—H26C	109.5
C4—C9—H9	119.8	H26B—C26—H26C	109.5
C13—S11—C11	89.62 (12)	C31—O31—H31O	106 (2)
O11—S12—O12	117.09 (11)	C32—N31—C33	117.09 (18)
O11—S12—N12	106.30 (11)	C32—N31—H31N	108.0
O12—S12—N12	112.52 (11)	C33—N31—H31N	108.0
O11—S12—C14	106.52 (11)	C32—N31—H32N	108.0
O12—S12—C14	107.51 (11)	C33—N31—H32N	108.0
N12—S12—C14	106.24 (11)	H31N—N31—H32N	107.3
C11—N11—C12	110.9 (2)	O31—C31—C32	111.9 (2)
C11—N12—S12	123.00 (17)	O31—C31—H31A	109.2
C17—N13—H11N	123 (2)	C32—C31—H31A	109.2
C17—N13—H12N	118 (2)	O31—C31—H31B	109.2
H11N—N13—H12N	117 (2)	C32—C31—H31B	109.2
N11—C11—N12	119.9 (2)	H31A—C31—H31B	107.9
N11—C11—S11	113.34 (18)	N31—C32—C31	110.55 (19)
N12—C11—S11	126.35 (18)	N31—C32—H32A	109.5
C13—C12—N11	116.3 (2)	C31—C32—H32A	109.5
C13—C12—H12	121.8	N31—C32—H32B	109.5
N11—C12—H12	121.8	C31—C32—H32B	109.5
C12—C13—S11	109.79 (19)	H32A—C32—H32B	108.1
C12—C13—H13	125.1	C36—C33—C34	110.7 (2)
S11—C13—H13	125.1	C36—C33—N31	105.70 (19)
C19—C14—C15	119.4 (2)	C34—C33—N31	109.4 (2)
C19—C14—S12	120.32 (18)	C36—C33—C35	109.8 (2)
C15—C14—S12	120.05 (19)	C34—C33—C35	112.3 (2)
C16—C15—C14	120.3 (2)	N31—C33—C35	108.62 (19)
C16—C15—H15	119.8	C33—C34—H34A	109.5
C14—C15—H15	119.8	C33—C34—H34B	109.5
C15—C16—C17	120.8 (2)	H34A—C34—H34B	109.5
C15—C16—H16	119.6	C33—C34—H34C	109.5
C17—C16—H16	119.6	H34A—C34—H34C	109.5
N13—C17—C18	121.5 (2)	H34B—C34—H34C	109.5
N13—C17—C16	120.6 (2)	C33—C35—H35A	109.5
C18—C17—C16	118.0 (2)	C33—C35—H35B	109.5
C19—C18—C17	121.1 (2)	H35A—C35—H35B	109.5
C19—C18—H18	119.4	C33—C35—H35C	109.5
C17—C18—H18	119.4	H35A—C35—H35C	109.5
C18—C19—C14	120.4 (2)	H35B—C35—H35C	109.5
C18—C19—H19	119.8	C33—C36—H36A	109.5
C14—C19—H19	119.8	C33—C36—H36B	109.5
C21—O21—H21O	107 (2)	H36A—C36—H36B	109.5
C22—N21—C23	117.87 (18)	C33—C36—H36C	109.5
C22—N21—H21N	107.8	H36A—C36—H36C	109.5
C23—N21—H21N	107.8	H36B—C36—H36C	109.5
			
O1—S2—N2—C1	47.6 (2)	S12—N12—C11—N11	−154.89 (18)
O2—S2—N2—C1	174.80 (18)	S12—N12—C11—S11	32.9 (3)
C4—S2—N2—C1	−69.7 (2)	C13—S11—C11—N11	0.25 (19)
C2—N1—C1—N2	−178.3 (2)	C13—S11—C11—N12	172.9 (2)
C2—N1—C1—S1	0.2 (3)	C11—N11—C12—C13	−0.4 (3)
S2—N2—C1—N1	179.38 (19)	N11—C12—C13—S11	0.6 (3)
S2—N2—C1—S1	1.1 (3)	C11—S11—C13—C12	−0.5 (2)
C3—S1—C1—N1	−0.5 (2)	O11—S12—C14—C19	−18.3 (2)
C3—S1—C1—N2	177.9 (2)	O12—S12—C14—C19	−144.61 (19)
C1—N1—C2—C3	0.3 (3)	N12—S12—C14—C19	94.7 (2)
N1—C2—C3—S1	−0.7 (3)	O11—S12—C14—C15	167.58 (19)
C1—S1—C3—C2	0.6 (2)	O12—S12—C14—C15	41.3 (2)
O1—S2—C4—C9	13.0 (2)	N12—S12—C14—C15	−79.4 (2)
O2—S2—C4—C9	−111.3 (2)	C19—C14—C15—C16	−0.6 (4)
N2—S2—C4—C9	134.9 (2)	S12—C14—C15—C16	173.55 (19)
O1—S2—C4—C5	−166.88 (19)	C14—C15—C16—C17	−0.5 (4)
O2—S2—C4—C5	68.8 (2)	C15—C16—C17—N13	−179.6 (2)
N2—S2—C4—C5	−45.0 (2)	C15—C16—C17—C18	1.5 (4)
C9—C4—C5—C6	0.8 (4)	N13—C17—C18—C19	179.5 (2)
S2—C4—C5—C6	−179.28 (18)	C16—C17—C18—C19	−1.6 (4)
C4—C5—C6—C7	−0.5 (4)	C17—C18—C19—C14	0.6 (4)
C5—C6—C7—N3	−177.7 (2)	C15—C14—C19—C18	0.5 (4)
C5—C6—C7—C8	−0.2 (3)	S12—C14—C19—C18	−173.60 (18)
N3—C7—C8—C9	178.1 (2)	C23—N21—C22—C21	171.49 (19)
C6—C7—C8—C9	0.6 (4)	O21—C21—C22—N21	55.5 (3)
C7—C8—C9—C4	−0.3 (4)	C22—N21—C23—C26	51.7 (3)
C5—C4—C9—C8	−0.4 (4)	C22—N21—C23—C24	170.4 (2)
S2—C4—C9—C8	179.69 (19)	C22—N21—C23—C25	−70.6 (3)
O11—S12—N12—C11	−155.40 (19)	C33—N31—C32—C31	175.3 (2)
O12—S12—N12—C11	−26.0 (2)	O31—C31—C32—N31	57.5 (3)
C14—S12—N12—C11	91.4 (2)	C32—N31—C33—C36	−177.4 (2)
C12—N11—C11—N12	−173.1 (2)	C32—N31—C33—C34	63.4 (3)
C12—N11—C11—S11	0.0 (3)	C32—N31—C33—C35	−59.5 (3)

### Hydrogen-bond geometry (Å, º)

**Table:**

*D*—H···*A*	*D*—H	H···*A*	*D*···*A*	*D*—H···*A*
O21—H21*o*···N1	0.84 (3)	1.90 (3)	2.739 (3)	177 (2)
O31—H31*o*···N2	0.83 (1)	1.97 (1)	2.796 (3)	174 (3)
N3—H2*n*···O12^i^	0.88 (2)	2.19 (2)	3.027 (3)	161 (3)
N13—H11*n*···O2^ii^	0.89 (2)	2.34 (2)	3.201 (3)	164 (2)
N13—H12*n*···O1^iii^	0.88 (2)	2.25 (2)	3.098 (3)	160 (2)
N21—H21*n*···N12	0.92	1.91	2.832 (3)	178
N21—H22*n*···O31	0.92	1.94	2.862 (3)	176
N31—H31*n*···N11	0.92	1.89	2.802 (3)	175
N31—H32*n*···O21	0.92	2.02	2.934 (3)	174
C2—H2···O11^iv^	0.95	2.43	3.356 (3)	164
C6—H6···O12^i^	0.95	2.50	3.263 (3)	137

Symmetry codes: (i) *x*, −*y*+3/2, *z*+1/2; (ii) −*x*+1, *y*+1/2, −*z*+1/2; (iii) *x*, −*y*+1/2, *z*−1/2; (iv) −*x*, *y*−1/2, −*z*+1/2.
